# Predictors of Health Literacy Levels in Patients Attending Anesthesia Outpatient Clinics for Preoperative Evaluation

**DOI:** 10.7759/cureus.51371

**Published:** 2023-12-30

**Authors:** Tuna Albayrak

**Affiliations:** 1 Anesthesiology and Reanimation, Giresun University, Faculty of Medicine, Giresun, TUR

**Keywords:** public healt, patient education, anesthesia outpatient clinic, preoperative evaluation, health literacy evaluation

## Abstract

Introduction: Health literacy significantly impacts healthcare outcomes, particularly in preoperative settings where patients' understanding of medical procedures, adherence to instructions, and surgical outcomes are influenced. Despite accessibility to medical information, challenges persist in comprehending healthcare details, affecting active patient participation in care. This study aims to assess health literacy among patients attending anesthesia outpatient clinics for preoperative evaluation and analyze associated factors.

Methods: A sample size of 240 patients was determined using power analysis. The inclusion criteria encompassed informed, consenting patients with adequate mental capacity and primary education, aged 18-65 years, according to the American Society of Anesthesiologists (ASA I-II). Data were collected through a descriptive characteristics form and the Health Literacy Scale (HLS).

Results: The mean health literacy score was 29.37±6.22, indicating a moderate level. Marital status, education level, employment status, book reading preference, comorbidities, and reason for clinic visits significantly influenced health literacy (p<0.001). Regression analysis revealed marital status (β=-1.915, p=0.047), employment (β=1.187, p=0.039), and book reading preference (β=3.76, p<0.001) as independent predictors of health literacy.

Discussion: Health literacy levels were associated with various socio-demographic and health-related factors. Notably, being single or divorced, employed, and enjoying reading predicted higher health literacy. This underscores the impact of social support, occupation, and reading habits on health literacy. However, longitudinal studies with objective measures are warranted to further explore these associations.

Conclusion: This study underscores the importance of addressing health literacy levels in anesthesia outpatient clinics, highlighting key predictors such as marital status, education, and employment. While contributing to our understanding of preoperative health literacy, further research using longitudinal designs and objective measures is essential. Bridging the health literacy gap is crucial for empowering patients, refining decision-making, and elevating the quality of overall surgical experiences.

## Introduction

Health literacy, an individual's ability to acquire, comprehend, and apply health-related information for informed decision-making, significantly shapes healthcare outcomes. In the realm of preoperative care, the health literacy level of patients attending anesthesia outpatient clinics profoundly impacts their understanding of medical procedures, adherence to preoperative instructions, and overall surgical outcomes [1].

This issue holds immense significance in public health, given the proven impact of interventions targeting health literacy on patient outcomes [2,3]. Patients awaiting surgical procedures often grapple with stress and anxiety, potentially leading to adverse consequences [4]. Despite anesthesia providers' efforts to deliver comprehensive explanations of anesthetic care plans, patient anxiety and time constraints can hinder the effective communication and assimilation of information [5]. Prior research has established a direct link between heightened patient knowledge of surgical information and reduced preoperative anxiety levels [6]. Alarmingly, a mere 32% of patients reported clear knowledge about anesthesia in a specific study, underscoring a significant knowledge gap likely rooted in an insufficient understanding of anesthesiologists' roles and anesthetic procedures [6]. Additionally, healthcare providers' perspectives might have a bigger impact on the informational quality and content shared with patients before surgery than the actual needs that patients perceive [7].

Despite advancements in medical technology and information accessibility, a substantial segment of the population faces challenges in grasping healthcare information, limiting their active engagement in their care. As patients gear up for surgery, their level of health literacy becomes increasingly critical, impacting their comprehension of anesthesia's risks and benefits, understanding of postoperative care instructions, and compliance with medication and dietary guidelines [8].

Understanding the health literacy rate among patients attending anesthesia outpatient clinics for preoperative evaluation is indispensable for tailoring communication strategies and enhancing patient understanding.

This study aims to delve into the health literacy levels of patients visiting anesthesia outpatient clinics for preoperative assessments. This study uses standard tests to find out how much people know about health and looks at the demographic and socioeconomic factors that are linked to those tests. The goal is to find out things that can help researchers come up with more effective ways to teach and communicate with people before surgery.

## Materials and methods

In the study, the sample size was determined using power analysis, with assumptions set at a study power of 99%, an alpha value of 0.05, and an effect size of 0.25. The G.Power program was utilized to calculate the sample size, resulting in a determined sample size of 240 patients for the study.

In the study, adhering to predefined inclusion and exclusion criteria was essential for the selection of patients for participation. Patients aged between 18 and 65 years, categorized as the ASA I-II group, who provided informed consent, possessed the mental capacity to comprehend survey questions, and had received at least primary school education, were evaluated for inclusion. Conversely, illiterate individuals who declined participation, had pathologies in the mental or central nervous system, had malignancies, or belonged to the ASA III-IV group were excluded from the study.

The data collection involved using both the Descriptive Characteristics Form and the Health Literacy Scale (HLS), facilitated through Google Forms. The Descriptive Characteristics Form comprises questions related to sociodemographic features, reading habits, accompanying illnesses, past surgeries, and various personal information. Meanwhile, the HLS was initially developed by Tuyen V. Duong et al. [9] in 2019 and was formatted as the Short Form Health Literacy Instrument, utilizing a 4-point Likert scale consisting of 12 items [10]. The mean score, calculated by dividing the total score by the number of items, was used in the formula Index = (mean-1) x 50/3 to evaluate health literacy. A higher index score indicated superior health literacy, within the possible range of 0 to 50.

The collected data underwent analysis using IBM Corp. Released 2013. IBM SPSS Statistics for Windows, Version 22.0. Armonk, NY: IBM Corp. The normality of the data distribution was assessed using the Kolmogorov-Smirnov test. Continuous variables were expressed as mean and standard deviation or interquartile range, contingent on their distribution. Categorical variables were presented as counts and percentages. Statistical analyses for continuous variables involved the application of the ‘Independent-Samples T test’, ‘Mann-Whitney-U test’, or ‘Kruskal-Wallis test’, as deemed appropriate. Post-hoc comparisons were conducted using the Dunnett T3 test. Correlation analyses between continuous variables were performed using either the Pearson correlation test or the Spearman rho correlation test based on assumptions. Additionally, multivariate linear regression analysis was employed to identify potential independent factors associated with the total health literacy score among patients. Throughout the analyses, the threshold for statistical significance was set at p < 0.05.

## Results

Among the 240 participants, 115 were male and 125 were female. There was no statistically significant difference in total health literacy scores between genders (p = 0.513). However, marital status showed a significant difference in health literacy scores (p<0.001), with married participants scoring lower compared to single or divorced individuals. Education level also significantly impacted health literacy scores (p<0.001), with those with primary education scoring lower than those with high school or university education (Table 1).

**Table 1:** Patient characteristics and health literacy Age (years) 50.33 ± 13.92, Mean Health Literacy Scor, mean ± sd: 29.37±6.22, minimum-maximum: 16.57-50 Data are presented as mean ± standard deviation, median (25-75 percentile), or n (%). Each similar superscript (a, b) indicates subsets of group categories with no statistically significant difference from each other at the p=0.05 level. * Independent T-test was used, ** Mann-Whitney-U test was used, *** Kruskal-Wallis test was used.

Characteristics		N (%)	Mean ± SD or Median (25-75 percentile)	p-value
Gender	Male	115 (47.9%)	29.09±6.39	0.513*
	Female	125 (52.1%)	29.62±6.09	
Marital status	Married	178 (74.2%)	27.78(23.61-31.94)^a^	<0.0013***
	Single	47 (19.6%)	30.56(29.17-33.33)^b^	
	Divorced	15 (6.3%)	36.11(33.33-36.11)^b^	
Education level	Primary	152 (63.3%)	27.78(23.61-31.94)^a^	<0.001***
	High school:	55 (22.9%)	33.33(25-37.5)b	
	Universty	33 (13.8%)	31.94(29.17-33.33)^b^	
Employment status	Not working	166 (69.2%)	28.38±5.59	0.001*
	Working	74 (30.8%)	31.59±7.01	
Economic Status	Good	35 (14.6%)	29.07±6.28	0.073*
	Moderate	205 (85.4%)	31.11±5.68	
Family Type	Nuclear	175 (72.9%)	29.66±6.38	0.238*
	Extended	65 (27.1%)	28.59±5.75	
Enjoy Reading Books	No	181 (75.4%)	27.93±5.56	<0.001**
	Yes	59 (24.6%)	33.78±6.13	
Any Comorbidity	No	119 (49.6%)	30.82±6.31	<0.001*
	Yes	121 (50.4%)	27.94±5.82	
Previous Surgery	No	119 (49.6%)	30.14±5.69	0.058*
	Yes	121 (50.4%)	28.62±6.64	
Current Visit for Surgery	No	216 (90%)	29.17(25-31.94)	<0.001**
	Yes	24 (10%)	34.03(31.94-39.58)	

Employment status was associated with higher health literacy scores (p = 0.001). There was no significant difference in health literacy scores concerning economic status (p = 0.073) or family type (p = 0.238). Participants who enjoyed reading books had significantly higher health literacy scores (p<0.001), as did those without comorbidities (p<0.001). Reasons for clinic visits also played a role, with those visiting for surgery showing higher health literacy scores (p<0.001) (Table 2).

**Table 2:** Health literacy survey questions Data shown as n (%)

Questions	Question Difficulty Level	n (%)
Finding information about the treatment of diseases that concern you is very difficult.	Very difficult	1 (0.4%)
	Quite difficult	80 (33.3%)
	Quite easy	143 (59.6%)
	Very easy	16 (6.7%)
Understanding the leaflets for medications (drug information leaflets) is very difficult.	Very difficult	2 (0.8%)
	Quite difficult	111 (46.3%)
	Quite easy	114 (47.5%)
	Very easy	13 (5.4%)
Understanding the leaflets for medications (drug information leaflets) is very difficult.	Very difficult	0 (0%)
	Quite difficult	91 (37.9%)
	Quite easy	122 (50.8%)
	Very easy	27 (11.3%)
Calling an ambulance in case of an emergency is very difficult.	Very difficult	0 (0%)
	Quite difficult	11 (4.6%)
	Quite easy	198 (82.5%)
	Very easy	31 (12.9%)
Access to information on how to manage mental health problems such as stress or depression.	Very difficult	3 (1.3%)
	Quite difficult	129 (53.8%)
	Quite easy	82 (34.2%)
	Very easy	26 (10.8%)
Understanding why you might need health screenings (such as breast exams, blood sugar tests, blood pressure checks) is very difficult.	Very difficult	0 (0%)
	Quite difficult	74 (30.8%)
	Quite easy	125 (52.1%)
	Very easy	41 (17.1%)
Deciding which vaccines, you might need is very difficult.	Very difficult	1 (0.4%)
	Quite difficult	123 (51.3%)
	Quite easy	79 (32.9%)
	Very easy	37 (15.4%)
Deciding how to protect yourself from illnesses based on advice from friends and family is very difficult.	Very difficult	0 (0%)
	Quite difficult	34 (14.2%)
	Quite easy	158 (65.8%)
	Very easy	48 (20%)
Gaining information about activities that improve mental health (meditation, exercise, walking, pilates, etc.).	Very difficult	5 (2.1%)
	Quite difficult	116 (48.3%)
	Quite easy	86 (35.8%)
	Very easy	33 (13.8%)
Understanding information from the media (internet, newspapers, magazines) about how to be healthier.	Very difficult	2 (0.8%)
	Quite difficult	55 (22.9%)
	Quite easy	132 (55%)
	Very easy	51 (21.3%)
Deciding which daily behaviors (drinking and eating habits, exercise, etc.) are related to your health.	Very difficult	1 (0.4%)
	Quite difficult	50 (20.8%)
	Quite easy	148 (61.7%)
	Very easy	41 (17.1%)
Joining a sports club or exercise activity.	Very difficult	19 (7.9%)
	Quite difficult	132 (55%)
	Quite easy	64 (26.7%)
	Very easy	25 (10.4%)

Regarding correlations, total health literacy scores were negatively correlated with marital status, age, comorbidities, and previous surgery (p<0.001 for all). Conversely, positive correlations were found between health literacy scores and education level, employment status, and enjoyment of reading books (p<0.001 for all). No significant correlations were found between health literacy scores and gender, economic status, or family type (Table 3).

**Table 3:** Relationship between health literacy score and patient characteristics *Spearman rho correlation test was used.  **Pearson correlation test was used.

Characteristic	R-Value	P-Value
Gender	0.072	0.263*
Marital Status	-0.307	<0.001*
Age (years)	-0.368	<0.001**
Education Level	0.332	<0.001*
Employment Status	0.209	0.001*
Economic Status	0.127	0.05*
Family Type	-0.071	0.274*
Interest in Reading Books	0.389	<0.001*
Presence of Any Additional Illness	-0.227	<0.001*
Previous Surgery Experience	-0.151	0.019*

A multivariable linear regression analysis was conducted to identify potential risk factors associated with health literacy. The analysis revealed a significant regression model (F(7, 232) = 11.19, p<0.001), explaining 23% of the variance in the dependent variable. Marital status (EXP (β): =-1.915, CI= -3.806 to -0.024), employment status (EXP (β): =1.187, CI= 0.059-2.316), and enjoyment of reading books (EXP (β): =3.76, CI= 1.941-5.579) were identified as independent parameters associated with health literacy (Table 4).

**Table 4:** Multivariate linear regression analysis of variables associated with health literacy CI: Confidence interval

Risk Factor	Expected (β) Value (95% CI)	P-Value
Marital Status (compared to being unmarried)	-1.915 (-3.806 to -0.024)	0.047
Age (years)	-0.032 (-0.091 to 0.028)	0.294
Education Level (compared to primary education)	1.187 (0.059 to 2.316)	0.039
Employment Status (compared to being unemployed)	1.443 (-0.207 to 3.094)	0.086
Interest in Reading Books (compared to not enjoying reading)	3.76 (1.941 to 5.579)	<0.001
Presence of Any Additional Illness (compared to having no additional illness)	0.259 (-1.449 to 1.966)	0.765
Previous Surgery Experience (compared to no previous surgery))	-0.537 (-1.984 to 0.91)	0.466

This summary condenses the main findings while preserving the key results and their significance in understanding the factors influencing health literacy among the study participants.

## Discussion

The aim of this study was to determine the health literacy levels of patients attending anesthesia outpatient clinics for preoperative evaluation and to examine the factors affecting health literacy. The results showed that the mean health literacy score of the participants was 29.37±6.22, indicating a moderate level of health literacy. The study also revealed that marital status, employment status, and enjoyment of reading books were significant predictors of health literacy, while gender, economic status, and family type were not.

The finding that marital status was negatively associated with health literacy was consistent with some previous studies by Chew et al. [11] and Mancuso [12] but contradicted others by Sayah and Williams [13] and Baker et al. [14]. A possible explanation for this discrepancy could be the cultural differences in the roles and responsibilities of married individuals in different societies. In some cultures, married individuals may have less time and opportunity to access and use health information due to variations in family obligations and social expectations worldwide. In contrast, in other cultures, married individuals may benefit from the support and guidance of their spouses and relatives in health-related matters.

The finding that employment status was positively associated with health literacy was in line with previous studies by Taş and Akış [15] and Mutlu et al. This could be attributed to the fact that employed individuals may have higher levels of education, income, and self-efficacy, which are known to influence health literacy [13]). Moreover, employed individuals may have more exposure to and access to health information through their work environment and social networks.

The finding that enjoyment of reading books was positively associated with health literacy was also consistent with previous studies by Garcia-Marcinkiewicz et al. [16] and Saltalı et al. [17]. This may be due to the fact that reading books can improve one's cognitive abilities, vocabulary, and comprehension, all of which are crucial for health literacy [13]. Furthermore, reading books may foster one's curiosity and interest in health topics and increase one's motivation to seek and use health information.

The finding that gender, economic status, and family type were not associated with health literacy was contrary to some previous studies by Wright et al. [18] and Demirel et al. [19]. A possible reason for this inconsistency could be the sample characteristics and the measurement tools used in different studies. For instance, some studies used different cut-off points or categories to define health literacy levels, which may affect the results and comparisons [20]. Additionally, some studies used different indicators or proxies to measure economic status and family type, which may not capture the complexity and diversity of these factors [15].

Study limitations: The study has some limitations that should be acknowledged. First, the study used a cross-sectional design, which limits the causal inference and the generalizability of the results. Second, the study used a self-report measure of health literacy, which may be subject to response bias and social desirability. Third, the study did not evaluate the patients' actual health behaviors and outcomes, which may depend on factors other than health literacy. Therefore, future studies should use longitudinal designs, objective measures, and clinical indicators to evaluate the health literacy of patients who come to the anesthesia outpatient clinic for preoperative evaluation.

## Conclusions

This study underscores the importance of addressing health literacy levels in anesthesia outpatient clinics, highlighting key predictors such as marital status, education, and employment. While contributing to our understanding of preoperative health literacy, further research using longitudinal designs and objective measures is essential. Bridging the health literacy gap is crucial for empowering patients, refining decision-making, and elevating the quality of overall surgical experiences.

## References

[1] Chang ME, Baker SJ, Dos Santos Marques IC (2020). Health literacy in surgery. Health Lit Res Pract.

[2] Berkman ND, Sheridan SL, Donahue KE (2011). Health literacy interventions and outcomes: an updated systematic review. Evid Rep Technol Assess (Full Rep).

[3] Bickmore TW, Pfeifer LM, Paasche-Orlow MK (2009). Using computer agents to explain medical documents to patients with low health literacy. Patient Educ Couns.

[4] Bryant MD, Schoenberg ED, Johnson TV, Goodman M, Owen-Smith A, Master VA (2009). Multimedia version of a standard medical questionnaire improves patient understanding across all literacy levels. J Urol.

[5] Campbell FA, Goldman BD, Boccia ML (2004). The effect of format modifications and reading comprehension on recall of informed consent information by low-income parents: a comparison of print, video, and computer-based presentations. Patient Educ Couns.

[6] Carbone ET, Lennon KM, Torres MI, Rosal MC (2005). Testing the feasibility of an interactive learning styles measure for U.S. Latino adults with type 2 diabetes and low literacy. Int Q Community Health Educ.

[7] Cordasco KM, Asch SM, Bell DS (2009). A low-literacy medication education tool for safety-net hospital patients. Am J Prev Med.

[8] Shahid R, Shoker M, Chu LM, Frehlick R, Ward H, Pahwa P (2022). Impact of low health literacy on patients' health outcomes: a multicenter cohort study. BMC Health Serv Res.

[9] Duong TV, Nguyen TT, Pham KM (2019). Validation of the short-form health literacy questionnaire (HLS-SF12) and ıts determinants among people living in rural areas in Vietnam. Int J Environ Res Public Health.

[10] Yılmaz Karahan S, Eskici G (2021). Sağlık okuryazarlığı ölçeği-kısa form ve dijital sağlıklı diyet okuryazarlığı ölçeğinin türkçe formunun geçerlik ve güvenirlik çalışması. İzmir Katip Çelebi Üniversitesi Sağlık Bilimleri Fakültesi Dergisi.

[11] Chew LD, Bradley KA, Boyko EJ (2004). Brief questions to identify patients with inadequate health literacy. Fam Med.

[12] Mancuso JM (2008). Health literacy: a concept/dimensional analysis. Nurs Health Sci.

[13] Sayah FA, Williams B (2012). An ıntegrated model of health literacy using diabetes as an exemplar. Canadian Journal of Diabetes.

[14] Baker DW, Wolf MS, Feinglass J, Thompson JA, Gazmararian JA, Huang J (2007). Health literacy and mortality among elderly persons. Arch Intern Med.

[15] Mutlu M, Mutlu A, Oktar D, Yelken B, Arslantas D, Unsal A (2022). Preoperative anxiety and health literacy in patients applying to the anesthesia outpatient clinic. Eur J Public Health.

[16] Garcia-Marcinkiewicz AG, Long TR, Danielson DR, Rose SH (2014). Health literacy and anesthesia: patients' knowledge of anesthesiologist roles and information desired in the preoperative visit. J Clin Anesth.

[17] Saltalı AÖ (2023). The effect of patients’ e-health literacy on their preoperative anxiety levels and fears about anesthesia. OPUS Jr Soc Res.

[18] Wright JP, Edwards GC, Goggins K (2018). Association of health literacy with postoperative outcomes in patients undergoing major abdominal surgery. JAMA Surg.

[19] Demirel A, Balkaya AN, Onur T, Karaca Ü, Onur A (2023). The effect of health literacy on preoperative anxiety levels in patients undergoing elective surgery. Patient Prefer Adherence.

[20] Jaime O, Suwei W, Macarthur E (2015). Preoperative patient education: Can we improve satisfaction and reduce anxiety?. Braz Jr Anes.

